# Update Lipidologie: Evidenzbasierte Behandlung von Fettstoffwechselstörungen

**DOI:** 10.1007/s00108-023-01536-8

**Published:** 2023-06-15

**Authors:** Klaus G. Parhofer

**Affiliations:** Medizinische Klinik IV – Großhadern, Klinikum der Universität München, Marchioninistr. 15, 81377 München, Deutschland

**Keywords:** Hypercholesterinämie, Hypertriglyzeridämie, Dyslipoproteinämie, Hyperlipoproteinämie, Lipoprotein(a), Hypercholesterolemia, Hypertriglyceridemia, Dyslipoproteinemia, Hyperlipoproteinemia, Lipoprotein(a)

## Abstract

Die Behandlung von erhöhten Lipidwerten spielt in der Atheroskleroseprävention eine zentrale Rolle, wobei der Low-Density-Lipoprotein(LDL)-Cholesterin-Senkung mit Statinen und, wenn nicht ausreichend, mit Ezetimib, Bempedoinsäure und Inhibitoren der Proproteinkonvertase Subtilisin/Kexin Typ 9 (PCSK9) die größte Bedeutung zukommt. Auch wenn Lebensstilmaßnahmen das kardiovaskuläre Risiko stark beeinflussen können, spielen sie für die Absenkung des LDL-Cholesterin-Werts nur eine untergeordnete Rolle. Für die Entscheidung, ob, und ggf. wie, intensiv eine Lipidsenkung durchgeführt werden soll, ist das kardiovaskuläre Absolutrisiko entscheidend. Die Zielwerte sind in den letzten Jahren aufgrund der Ergebnisse von Interventionsstudien weiter abgesenkt worden. Bei Patienten mit sehr hohem Risiko (z. B. Patienten mit manifester Atheroskleroseerkrankung) sollten ein LDL-Cholesterin-Wert < 55 mg/dl (< 1,4 mmol/l; Umrechnung: [mg/dl] • 0,02586 = [mmol/l]) und mindestens eine Halbierung des Ausgangswertes angestrebt werden. Hinsichtlich erhöhter Triglyzeridwerte, entweder isoliert oder bei gleichzeitiger Erhöhung des LDL-Cholesterin-Werts, sind die Behandlungsziele weniger klar definiert, obwohl auch erhöhte Triglyzeridspiegel kausal mit Atheroskleroseereignissen verknüpft sind. Lebensstilmaßnahmen können die Triglyzeridspiegel deutlich absenken und sind oft effektiver als spezifische Triglyzeridsenker wie Fibrate und Omega-3-Fettsäuren. Neue Lipidsenker zur Behandlung bei stark erhöhten Triglyzerid- und erhöhten Lipoprotein(a)Werten sind in der Entwicklung, müssen ihren Nutzen aber erst in Endpunktstudien belegen.

## Abstract (en)

The treatment of elevated plasma lipid levels plays an important role in prevention of atherosclerosis. Lowering of low-density lipoprotein (LDL) cholesterol with statins and if required with additional ezetimibe, bempedoic acid and proprotein convertase subtilisin/kexin type 9 (PCSK9) inhibitors is of utmost importance. While lifestyle modification can strongly influence the cardiovascular risk, it only plays a minor role in lowering LDL cholesterol values. The overall (absolute) cardiovascular risk determines if and in what intensity lipid-lowering treatment should be implemented. Based on new results from interventional studies the LDL cholesterol target values have been reduced in recent years. Thus, in patients with a very high risk (for example patients with established atherosclerotic disease) an LDL cholesterol level of < 55 mg/dl (< 1.4 mmol/l, conversion mg/dl×0.02586=mmol/l) and at least a 50% reduction from baseline should be strived for. With respect to elevated triglyceride levels, either alone or simultaneously with elevated LDL cholesterol levels, the treatment goals are less clearly defined, despite the fact that elevated triglyceride levels are causally linked to atherosclerotic events. Lifestyle modifications can significantly reduce triglyceride levels and are often more effective than specific triglyceride-lowering medications, such as fibrates and omega‑3 fatty acids. New lipid-lowering drugs for the treatment of patients with severely elevated triglyceride levels and elevated lipoprotein(a) levels are being developed but their clinical benefits still have to be confirmed in endpoint studies.

Lipidstoffwechselstörungen sind in vielen Bereichen der Medizin relevant. Freie Fettsäuren spielen als Mediatoren bei Entzündungen, aber auch in onkologischen Prozessen eine wichtige Rolle, stehen im Zusammenhang mit neurodegenerativen Erkrankungen (über Apolipoprotein E4) und sind in der Diabetologie wichtig [1]. Einerseits weisen viele Patienten mit einem Diabetes mellitus eine für das Atheroskleroserisiko entscheidende Fettstoffwechselstörung auf, andererseits spielen Lipidveränderungen auch in der Genese des Diabetes mellitus Typ 2 eine wichtige Rolle. Schließlich kann eine ausgeprägte Hypertriglyzeridämie eine akute Pankreatitis auslösen [2].

Klinisch am wichtigsten ist allerdings der kausale Zusammenhang zwischen einer erhöhten Plasmalipidkonzentration und Atherosklerose und damit kardiovaskulären Ereignissen. Dieser Zusammenhang ist epidemiologisch gesichert, pathophysiologisch geklärt und durch überzeugende Endpunktstudien validiert [3]. Wenige Ansätze in der Inneren Medizin sind so gut durch hochrangige Endpunktstudien belegt wie die Prävention kardiovaskulärer Erkrankungen durch Lipidsenker.

## Diagnostik

Die inzwischen praktisch ubiquitär durchgeführte direkte Bestimmung des Low-Density-Lipoprotein(LDL)-Cholesterin-Werts erlaubt die Untersuchung der Lipidwerte im nichtnüchtern Blut. Lediglich bei Hypertriglyzeridämien sollte weiterhin eine Nüchternblutentnahme erfolgen, da Triglyzeridkonzentrationen postprandial sehr stark schwanken können [4]. Daneben ist eine Nüchternblutabnahme notwendig, wenn die Bestimmung des LDL-Cholesterin-Werts über die sog. Friedewald-Formel erfolgt.

Der Lipidstatus sollte folgende Parameter umfassen, um eine adäquate Abschätzung der Behandlungsbedürftigkeit durchführen zu können:

- Gesamtcholesterin,
- Triglyzeride,
- LDL-Cholesterin,
- High-Density-Lipoprotein(HDL)-Cholesterin,
- Non-HDL-Cholesterin (berechnet),
- Lipoprotein(a) (einmalig),

Weitere Parameter sind Spezialsituationen vorbehalten, und ihre Bestimmungen führen meist zu keiner therapeutischen oder prognostischen Änderung. Erwähnenswert sind in diesem Zusammenhang Messungen der Apolipoprotein-B(ApoB)-Konzentration, die, wie eine Reihe von epidemiologischen Auswertungen zeigt, das kardiovaskuläre Risiko besser abschätzen lassen als LDL-Cholesterin-Messungen [5]. Allerdings gibt es derzeit keine Empfehlung, ApoB-Messungen routinemäßig durchzuführen. Eine Bestimmung des LDL-HDL-Quotienten bzw. des Gesamtcholesterin-HDL-Quotienten wird nicht mehr empfohlen Dies spiegelt wider, dass höhere HDL-Cholesterin-Werte nur bis zu einer gewissen Konzentration protektiv wirken und dass keine Linearität besteht [6].

Im Erwachsenenalter sollten die Lipidwerte alle 3 bis 5 Jahre überprüft werden, oder falls sich neue wesentliche Begleiterkrankungen, wie Diabetes mellitus, Nierenerkrankungen oder Adipositas, ergeben. Bei Kindern wird der Lipidstatus derzeit nur bei Verdacht auf eine familiäre Hypercholesterinämie oder bei anderen wesentlichen Begleiterkrankungen, wie Diabetes mellitus, ermittelt. Nach einer Therapieveränderung sollte der Lipidstatus nach 4 bis 6 Wochen kontrolliert werden, dann nach 3 Monaten und schließlich in 3‑ bis 6‑monatigem Abstand.

Von großer Bedeutung ist die relativ große Messungenauigkeit von LDL-Cholesterin v. a. bei niedrigen Werten. Zwischen einzelnen Laboren gibt es erhebliche Unterschiede, sodass es sinnvoll ist, bei Serienmessungen die Werte immer im selben Labor bestimmen zu lassen. Dies ist insbesondere relevant, wenn die strengen von der European Society of Cardiology (ESC) geforderten Zielwerte umgesetzt werden sollen. Hier sollte man sich vor Augen halten, dass aufgrund der Messungenauigkeit ein LDL-Cholesterin-Wert von 65 mg/dl (1,7 mmol/l; Umrechnung: [mg/dl] • 0,02586=[mmol/l]) nicht sicher von einem Wert von 55 mg/dl (1,4 mmol/l) zu unterscheiden ist [7].

### Merke.

- Nüchternbestimmungen sind v. a. bei vermuteter oder bekannter Hypertriglyzeridämie notwendig, ansonsten können Nichtnüchternwerte verwendet werden.
- Der Lipidstatus sollte Cholesterin, HDL-Cholesterin, LDL-Cholesterin, Non-HDL-Cholesterin (berechnet), Triglyzeride und Lipoprotein(a) (einmalig) umfassen.

## Einteilung

In der Praxis hat sich eine rein klinische Einteilung (Tab. 1) durchgesetzt, da andere Einteilungen (aufgrund der Genetik oder die Klassifikation nach Fredrickson [8]) klinisch viele Aspekte unberücksichtigt lassen. Zu beachten ist, dass eine Konzentrationserhöhung des Lipoprotein(a) isoliert oder in Kombination mit jeder anderen Fettstoffwechselstörung auftreten kann. Die familiäre Dysbetalipoproteinämie ist eine Sonderform der gemischten Hyperlipoproteinämie und die familiäre Hypercholesterinämie der Prototyp der LDL-Hypercholesterinämie.

**Table 1:** 

Fettstoffwechselstörung	Cholesterin	Triglyzeride	LDL-Cholesterin	HDL-Cholesterin	Non-HDL-Cholesterin
LDL-Hypercholesterinämie	↑	–	↑	–	↑
Hypertriglyzeridämie	↑	↑	–	↓	↑
Gemischte Hyperlipoproteinämie	↑	↑	↑	↓	↑
HDL-Erniedrigung	–	–	–	↓	(↑)
Lipoprotein(a) Erhöhung	Kann isoliert oder zusammen mit oben genannten Fettstoffwechselstörungen auftreten				

*HDL* High Density Lipoproteins, *LDL* Low Density Lipoproteins

## Korrelationen mit kardiovaskulären Erkrankungen

Nach heutigem Verständnis ist die Konzentration ApoB-haltiger Lipoproteine die entscheidende Determinante für das Auftreten kardiovaskulärer Erkrankungen. Dabei ist zu beachten, dass LDL, aber auch Lipoprotein(a) sowie eine Reihe von mehr oder weniger triglyzeridreichen Remnant-Partikeln ApoB enthalten und somit atherogen sind. Bei Patienten ohne Hypertriglyzeridämie korreliert der LDL-Cholesterin-Spiegel sehr gut mit der ApoB Konzentration und erlaubt eine adäquate Abschätzung des Atheroskleroserisikos. Bei Patienten mit gemischter Hyperlipoproteinämie oder Hypertriglyzeridämie besteht jedoch meist eine Diskrepanz zwischen der ApoB-Konzentration und dem LDL-Cholesterin-Spiegel, sodass der Non-HDL-Cholesterin- oder der ApoB-Spiegel das kardiovaskuläre Risiko besser darstellt.

### Gesamtcholesterin

Der Gesamtcholesterinspiegel korreliert mit kardiovaskulären Erkrankungen. Dies spiegelt im Wesentlichen wider, dass LDL-Cholesterin die entscheidende Determinante für das Gesamtcholesterin darstellt.

### Low-Density-Lipoprotein-Cholesterin

Der LDL-Cholesterin-Spiegel korreliert sehr gut mit dem Atheroskleroserisiko. Allerdings muss beachtet werden, dass bei Patienten mit gemischter Hyperlipoproteinämie (z. B. bei Diabetes mellitus) häufig sog. kleine, dichte LDL-Partikel vorliegen. In diesem Fall würden durch die Betrachtung der LDL-Cholesterin-Konzentration die Zahl der Apo-B-haltigen Lipoproteine und auch das Atheroskleroserisiko unterschätzt.

### Lipoprotein(a)

Lipoprotein(a) ist ein LDL-artiges Partikel, das durch das zusätzliche Apoprotein(a) auch thrombogene Eigenschaften erhält. Erhöhte Lipoprotein(a)-Konzentrationen sind mit Atherosklerose und Aortenklappenstenose assoziiert, nicht aber mit venösen Thromben. Die Lipoprotein(a)-Konzentration ist weitgehend genetisch determiniert; deshalb wird nur eine einmalige Messung empfohlen [9, 10].

### Triglyzeride

Die Konzentration triglyzeridreicher Lipoproteine korreliert ebenfalls mit dem kardiovaskulären Risiko [11]. Allerdings besteht eine erhebliche Variabilität, da bei steigenden Triglyzeridkonzentrationen nicht unbedingt die Zahl der Partikel zunimmt, sondern evtl. nur deren Beladung mit Triglyzeriden. In diesem Fall kann davon ausgegangen werden, dass das kardiovaskuläre Risiko nicht so stark erhöht ist. So ist auch zu erklären, warum das kardiovaskuläre Risiko bis zu Triglyzeridwerten von ca. 400 mg/dl (4,5 mmol/l; Umrechnung: [mg/dl] • 0,01129 = [mmol/l]) kontinuierlich ansteigt, danach mit steigenden Triglyzeridwerten aber nicht mehr wesentlich höher wird [11].

### High-Density-Lipoprotein-Cholesterin

Während man früher davon ausging, dass der HDL-Cholesterin-Spiegel kausal mit der Atherosklerose im Zusammenhang steht, sprechen genetische Daten und Ergebnisse von Interventionsstudien inzwischen gegen eine Kausalität [6, 12]. Es scheint, dass der HDL-Cholesterin-Spiegel einen Marker für die eigentlich atherogenen triglyzeridreichen Lipoproteine darstellt. Unabhängig davon, spielen allerdings HDL-Partikel im Atheroskleroseprozess auch direkt eine Rolle, da sie nicht nur den Cholesterinefflux regulieren können, sondern auch andere pro- und antiatherogene Signale transportieren können. Diese Funktion spiegelt sich aber nur sehr begrenzt in der HDL-Cholesterin Konzentration wider.

Als Marker für das Atheroskleroserisiko ist der HDL-Cholesterin-Spiegel weiter wichtig: Personen mit höheren HDL-Cholesterin-Werten haben ein niedrigeres kardiovaskuläres Risiko als solche mit niedrigen Werten. Allerdings scheint dies nur bis zu einer HDL-Cholesterin-Konzentration von ca. 70 mg/dl (1,8 mmol/l) der Fall zu sein. Noch höhere HDL-Cholesterin-Spiegel sind nicht mit einer geringeren Atheroskleroserate assoziiert. Im Gegenteil, bei noch höheren HDL-Cholesterin-Spiegeln scheint die Atheroskleroserate wieder zu steigen [6].

### Non-High-Density-Lipoprotein-Cholesterin

Non-HDL-Cholesterin (Gesamtcholesterin minus HDL-Cholesterin) spiegelt die Konzentration aller ApoB-haltigen Lipoproteine (das ApoB des „armen Mannes“) wider und ist, insbesondere bei vorliegender Hypertriglyzeridämie ein besserer Marker für die Atherogenität des Lipidstatus als der LDL-Cholesterin-Spiegel.

## Zielwerte

Überlegungen zur Notwendigkeit einer lipidsenkenden Therapie werden praktisch immer unter dem Aspekt der Prävention kardiovaskulärer Erkrankungen angestellt. Bei exzessiven Hypertriglyzeridämien (> 1000 mg/dl bzw. ca. 10 mmol/l) spielt auch die Pankreatitisprävention eine Rolle [2].

Es steht zweifelsfrei fest, dass durch eine Absenkung des LDL-Cholesterin-Werts kardiovaskuläre Ereignisse verhindert werden können [3]. Dies betrifft nach derzeitigem Kenntnisstand jede Form der LDL-Cholesterin-Wert-Senkung, wurde jedoch in entsprechenden Endpunktstudien bisher für die Therapie mit Statinen, Ezetimib, Bempedoinsäure, Inhibitoren der Proproteinkonvertase Subtilisin/Kexin Typ 9 (PCSK9) und begrenzt auch mit Gallensäurebinder gezeigt. Eine absolute LDL-Cholesterin-Wert-Senkung überträgt sich in eine relative Risikoreduktion. Aufgrund der derzeitigen Datenlage kann davon ausgegangen werden, dass eine LDL-Cholesterin-Wert-Senkung um 1 mmol/l (ca. 40 mg/dl) das relative Risiko um 20–22 % reduziert. Dies trifft auf Niedrigrisikopopulationen ebenso zu wie auf Hochrisikopopulationen. Eine LDL-Cholesterin-Wert-Senkung ist dann besonders effektiv (niedrige „numbers needed to treat“, große Anzahl verhinderter Ereignisse), wenn der LDL-Cholesterin-Wert bei einem hohen Ausgangsrisiko stark gesenkt wird [13]. Unter Berücksichtigung dieser Fakten definieren inzwischen praktisch alle Fachgesellschaften verschiedene Risikogruppen, die einer unterschiedlich intensiven (oder keiner) Lipidsenkung zugeführt werden. Gewisse Unterschiede gibt es in der Umsetzung dieser Überlegungen. Während die europäischen Empfehlungen aus dem Jahr 2019 (Tab. 2) sehr stark auf das Erreichen bestimmter LDL-Cholesterin-Zielwerte fokussieren (bei gleichzeitiger Absenkung des LDL-Cholesterin-Ausgangswerts um 50 % oder mehr), rücken die amerikanischen Empfehlungen die relative LDL-Cholesterin-Wert-Senkung stärker in den Vordergrund [3, 14]. Allerdings haben inzwischen auch die amerikanischen Empfehlungen (wieder) einen LDL-Cholesterin-Zielwert für Patienten mit sehr hohem Risiko aufgenommen (< 70 mg/dl, 1,8 mmol/l).

**Table 2:** 

	LDL-Cholesterin^a^	Non–HDL-Cholesterin^a^	Apolipoprotein B^b^
	Primärer Zielwert	Sekundäre Zielwerte	
*Sehr hohes Risiko* Nachgewiesene KHK oder andere Atherosklerosemanifestation, Typ-1- oder Typ-2-Diabetes mit Endorganschaden oder ≥ 3 RF, chronische Niereninsuffizienz (GFR < 30 ml/min), Zehnjahresrisiko ≥ 10 % (SCORE)	< 55 mg/dl (< 1,4 mmol/l) und ≥ 50 %ige Absenkung vom Ausgangswert	< 85 mg/dl (< 2,2 mmol/l)	< 65 mg/dl (1,2 µmol/l)
*Hohes Risiko* Deutlich erhöhte RF wie bei FH, schwerem Hypertonus oder Diabetes mellitus oder Zehnjahresrisiko ≥ 5 bis < 10 % (SCORE)	< 70 mg/dl (< 1,8 mmol/l) und ≥ 50 %ige Absenkung vom Ausgangswert	< 100 mg/dl (< 2,6 mmol/l)	< 80 mg/dl (1,5 µmol/l)
*Moderates Risiko* Diabetes mellitus < 10 J, keine RF/Endorganschaden; Zehnjahresrisiko ≥ 1 bis < 5 % (SCORE)	< 100 mg/dl (< 2,6 mmol/l)	< 130 mg/dl (< 3,4 mmol/l)	Nicht definiert
*Geringes Risiko* Zehnjahresrisiko < 1 % (SCORE)	< 115 mg/dl (< 3,0 mmol/l)	< 145 mg/dl (< 3,8 mmol/l)	Nicht definiert

Das SCORE-Risiko bezieht sich auf das Zehnjahresrisiko für ein tödliches kardiovaskuläres Ereignis (www.scardio.org); bei Patienten mit besonders hohem Risiko (rezidivierendes Ereignis innerhalb von 2 Jahren trotz maximaler Statintherapie) kann ein LDL-Cholesterin-Zielwert < 40 mg/dl (< 1,0 mmol/l) erwogen werden (modifiziert nach Mach et al. [3])

*FH* familiäre Hypercholesterinämie, *GFR* glomeruläre Filtrationsrate, *HDL* High Density Lipoproteins, *KHK* koronare Herzkrankheit, *LDL* Low Density Lipoproteins, *RF* Risikofaktor

^a^Cholesterin: Umrechnung: [mg/dl] • 0,02586 = [mmol/l]

^b^Apolipoprotein B: Umrechnung: [mg/dl] • 0,0182 = [µmol/l]

Die früher und auch heute noch von manchen Fachgesellschaften propagierte „Fire-and-forget“-Strategie wird inzwischen sehr kritisch gesehen, da sie zu einer Fehlversorgung führt und mit einer schlechteren Adhärenz verknüpft ist. Auch konnte gezeigt werden, dass „fire and forget“ im Vergleich zu einer zielwertorientierten Therapie mit einem schlechteren Outcome verknüpft ist [15].

### Merke.

- Primärer Zielparameter ist der LDL-Cholesterin-Spiegel.
- Das Non-HDL-Cholesterin und ApoB stellen sekundäre Zielparameter dar und sind v. a. bei kombinierter Hyperlipoproteinämie oder Hypertriglyzeridämie wichtig.
- Das HDL-Cholesterin ist kein Zielparameter.

## Therapieansätze

### Lebensstilmaßnahmen

Lebensstilmaßnahmen spielen in der Prävention kardiovaskulärer Erkrankungen eine enorme Rolle. Der langfristige Effekt von Lebensstilmaßnahmen ist in der Summe fast so groß wie die Summe der genetischen Faktoren. In einer 2016 publizierten Auswertung wurde gezeigt, dass ein gesunder Lebensstil bei „ungünstiger“ genetischer Ausstattung das kardiovaskuläre Risiko in etwa halbieren kann, und dass dieses in einen Bereich von Personen mit „günstiger“ genetischer Ausstattung gesenkt werden kann [16]. Ein günstiger Lebensstil war lediglich durch 4 Faktoren (Normalgewicht, Rauchverzicht, mediterrane Kost und moderate körperliche Aktivität) charakterisiert.

Lebensstilmaßnahmen beeinflussen aber auch die Lipidwerte direkt. Besonders ausgeprägt ist der Effekt auf den Triglyzeridspiegel und auf das HDL-Cholesterin. Damit verändert sich auch Non-HDL-Cholesterin günstig. Dagegen ist der Effekt auf LDL-Cholesterin eher gering und auf Lipoprotein(a) fehlend. Die Beeinflussung von Lebensstilmaßnahmen auf den Lipidstoffwechsel ist in Tab. 3 zusammengefasst.

**Table 3:** 

Fettstoffwechselstörung	Lebensstilmaßnahmen	Effektivität
LDL-Hypercholesterinämie	Reduktion der Zufuhr tierischer Fette Reduktion der Cholesterinzufuhr (minimaler Effekt) Steigerung körperlicher Aktivität	Nur geringe Absenkung von LDL-Cholesterin (5–10 %) Bei gemischter Hyperlipoproteinämie teilweise stärkeres Ansprechen
Hypertriglyzeridämie/kombinierte Hyperlipoproteinämie	Reduktion/Verzicht auf Alkohol Reduktion schnell verstoffwechselbarer Kohlenhydrate Reduktion der Zufuhr tierischer Fette Steigerung der körperlichen Aktivität	Ansprechen sehr variabel Teilweise gutes oder sehr gutes Ansprechen (Absenkung der Triglyzeridwerte um über 70 %, teilweise Normalisierung)
Lipoprotein(a)-Erhöhung	Keine spezifischen Maßnahmen	Lässt sich durch Lebensstilmaßnahmen kaum beeinflussen

*LDL* Low Density Lipoproteins

### Medikamentöse Therapie

Eine zusammenfassende Darstellung der medikamentöse Therapieansätze findet sich in Tab. 4.

**Table 4:** 

Ansatz	Lipideffekt	Kommentar
Statine (Atorvastatin, Fluvastatin, Pitavastatin, Pravastatin, Rosuvastatin, Simvastatin)	LDL-Cholesterin: ↓↓-↓↓↓ HDL-Cholesterin: ↑ Triglyzeride: ↓	Unter hoher Dosierung LDL-Cholesterin-Wert-Reduktion um bis zu 55 % möglich; zahlreiche positive Endpunktstudien; relative Risikoreduktion korreliert mit LDL-Wert-Senkung (22 %ige relative Risikosenkung/mmol LDL-Cholesterin-Wert-Absenkung)
Ezetimib	LDL-Cholesterin ↓↓ HDL-Cholesterin: neutral Triglyzeride: neutral	In Kombination mit Statinen LDL-Cholesterin-Wert-Senkung 20–25 %; Endpunktstudie derzeit nur in Kombination mit Simvastatin vorhanden
Bempedoinsäure	LDL-Cholesterin ↓↓ HDL-Cholesterin: neutral Triglyzeride: neutral	In Kombination mit Ezetimib LDL-Cholesterin-Wert-Senkung 36 %; Endpunktstudie bei Statin-intoleranten Patienten vorhanden
PCSK9-Inhibitoren (Alirocumab, Evolocumab, Inclisiran)	LDL-Cholesterin: ↓↓↓ HDL-Cholesterin: ↑ Triglyzeride: ↓ Lipoprotein(a): ↓	Allein und in Kombination mit anderen Lipidsenkern LDL-Cholesterin-Wert-Reduktion um 50–60 %; positive Endpunktstudien (in Kombination mit Statinen) zu Alirocumab und Evolocumab (PCSK9-Antikörper); Risikoreduktion korreliert mit LDL-Wert-Senkung; Patienten mit höchstem Risiko profitieren am meisten; bisher keine Endpunktstudie zu Inclisiran (si-RNA)
Gallensäurebinder (Colestyramin, Colesevelam)	LDL-Cholesterin: ↓↓ HDL-Cholesterin: ↑ Triglyzeride: ↑‑↑↑	In Kombination mit Statinen LDL-Wert-Senkung um bis zu 30 % möglich; ausgeprägtes Nebenwirkungsprofil; ältere Endpunktstudie zeigt positiven Effekt auf Myokardinfarktrate und Gesamtmortalität [17]
Fibrat	LDL-Cholesterin: ↓ HDL-Cholesterin: ↑ Triglyzeride: ↓↓-↓↓↓	Bei Hypertriglyzeridämie oder kombinierter Hyperlipoproteinämie teilweise deutliche Triglyzerid-Wert-Reduktion; ältere Endpunktstudien positiv; Endpunktstudien in Kombination mit Statinen zeigen keine Risikoreduktion
Omega-3-Fettsäuren	LDL-Cholesterin: neutral HDL-Cholesterin: ↑ Triglyzeride: ↓↓	Bei höherer Dosis (2–4 g/Tag) teilweise deutliche Triglyzerid-Wert-Absenkung; Endpunktstudien mit geringer Dosis (1 g/Tag) in Kombination mit Statinen enttäuschend; eine Endpunktstudie bei statinbehandelten Hochrisikopatienten unter Verwendung von 4 g EPA positiv
Volanesorsen	LDL-Cholesterin: neutral HDL-Cholesterin: ↑ Triglyzeride: ↓↓	Nur bei genetisch bestätigtem familiären Chylomikronämie-Syndrom und hohem Risiko für Pankreatitis (rezidivierender akuter Pankreatitis)
Evinacumab	LDL-Cholesterin: ↓↓↓ HDL-Cholesterin: ↓ Triglyzeride: ↓↓	Nur bei genetisch gesicherter homozygoter familiärer Hypercholesterinämie
Lomitapid	LDL-Cholesterin: ↓↓↓ HDL-Cholesterin: neutral Triglyzeride: ↓	Nur bei genetisch gesicherter homozygoter familiärer Hypercholesterinämie

*EPA* Eicosapentaensäure, *HDL* High Density Lipoproteins, *LDL* Low Density Lipoproteins, *PCSK9* Proproteinkonvertase Subtilisin/Kexin Typ 9, *si-RNA* „small interfering ribonucleic acid“

#### Statine

Statine senken den LDL-Cholesterin-Spiegel um über 50 % (in hoher Dosierung). Zahlreiche Studien und Metaanalysen haben gezeigt, dass sich die statinvermittelte LDL-Cholesterin-Wert-Senkung in eine kardiovaskuläre Risikoreduktion überträgt, wobei der Nutzen weitgehend proportional zum Ausmaß der LDL-Wert-Senkung ist [18]. Ob Statine einen protektiven Effekt haben, der über die LDL-Wert-Senkung hinausgeht, ist weiter unklar. Statine werden in der Mehrzahl der Fälle gut vertragen, bei ca. 10 % der Patienten treten jedoch Muskelbeschwerden auf (ohne und selten mit Konzentrationserhöhung der Kreatinkinase [CK]). Daneben können Leberwerterhöhungen vorkommen (ca. 2 %); Leberversagen ist extrem selten (wenige Einzelfälle). Die einzelnen Statine unterscheiden sich in ihrer Wirksamkeit (am wirksamsten sind Atorvastatin und Rosuvastatin) und in ihrer Nebenwirkungsrate (am besten verträglich Pravastatin und Fluvastatin). Aufgrund der überzeugenden Datenlage sind Statine Lipidsenker der 1. Wahl, wenn eine medikamentöse lipidsenkende Therapie indiziert ist.

#### Ezetimib

Ezetimib ist ein Cholesterinadsorptionshemmer, der den LDL-Cholesterin-Wert insbesondere in Kombination mit Statinen deutlich absenkt (ca. minus 20 %). Eine große Endpunktstudie zeigt, dass sich die ezetimibbedingte Absenkung des LDL-Cholesterin-Werts ebenfalls in eine kardiovaskuläre Risikoreduktion überträgt, wobei das Ausmaß der kardiovaskulären Risikoreduktion dem Ausmaß der beobachteten LDL-Cholesterin-Wert-Senkung entspricht [19]. Ezetimib kommt v. a. zum Einsatz, wenn mittlere oder hohe Dosen von Statinen nicht ausreichen, um dem LDL-Cholesterin-Zielwert zu erreichen. Daneben wird Ezetimib Patienten mit Statinintoleranz allein oder in Kombination mit niedrigen Statindosen verabreicht.

#### Bempedoinsäure

Bempedoinsäure ist ein Pro-Drug und bedarf der Aktivierung durch das in Leberzellen exprimierte Enzym Acyl-CoA-Synthetase. Das aktivierende Enzym ist in Skelettmuskelzellen nicht vorhanden, sodass Bempedoinsäure nicht zu muskelbezogenen Nebenwirkungen, wie sie von Statinen bekannt sind, führt. Der aktive Metabolit (ETC-1002-CoA) hemmt die Adenosintriphosphat-Zitrat-Lyase (ACL) und die hepatische Cholesterinsynthese auf einer früheren Stufe als Statine. In der Monotherapie wird eine LDL-Cholesterin-Wert-Senkung um ca. 20 % beobachtet, in Kombination mit Ezetimib eine Absenkung um 36 %. Auch in Kombination mit Statinen ergibt sich eine zusätzliche LDL-Cholesterin-Wert-Senkung. An Nebenwirkungen sind eine Erhöhung der Harnsäurekonzentration sowie eine erhöhte Cholelithiasisrate zu nennen. Im Rahmen einer Endpunktstudie an statinintoleranten Patienten konnte gezeigt werden, dass sich die bempedoinsäurebedingte Absenkung des LDL-Cholesterin-Werts ebenfalls in eine kardiovaskuläre Risikoreduktion überträgt, wobei das Ausmaß der kardiovaskulären Risikoreduktion dem Ausmaß der beobachteten LDL-Cholesterin-Wert-Senkung entspricht [20]. Bempedoinsäure wird derzeit v.a. in 2 Situationen eingesetzt: einerseits bei statinintoleranten Patienten und andererseits bei Patienten, die trotz einer Statin-Ezetimib-Kombination die Zielwerte nicht erreichen.

#### Inhibitoren der Proproteinkonvertase Subtilisin/Kexin Typ 9

Die PCSK9-Inhibitoren umfassen PCSK9-Antikörper (Alirocumab und Evolocumab) sowie die „small interfering ribonucleic acid“ (si-RNA) Inclisiran. Sie können eine zusätzliche LDL-Cholesterin-Wert-Senkung um 50–60 % induzieren und wirken in Kombination mit anderen Lipidsenkern, aber auch in Monotherapie. Die PCSK9-Antikörper (Alirocumab und Evolocumab) werden 2‑wöchentlich oder 4‑wöchentlich s.c. appliziert, Inclisiran erneut nach 3 Monaten, dann 6‑monatlich. In 2 großen Endpunktstudien konnte belegt werden, dass sich die Therapie mit Alirocumab und Evolocumab in eine kardiovaskuläre Risikoreduktion überträgt [21, 22]. Zahlreiche Subauswertungen machen deutlich, dass die relative und absolute Risikoreduktion umso größer sind, je höher das Absolutrisiko ist [23]. Für Inclisiran liegen diese Daten bisher nicht vor, da die entsprechende Endpunktstudie noch nicht abgeschlossen ist (Abschluss voraussichtlich 2025). Die PCSK9-Inhibitoren spielen insbesondere bei Patienten mit sehr hohem Risiko und Nichterreichen der Zielwerte unter einer Therapie mit Statinen plus Ezetimib plus evtl. Bempedoinsäure eine wichtige Rolle [24]. Sie werden von den meisten Patienten mit einer Statinintoleranz gut vertragen. Limitierend für den Einsatz von PCSK9-Inhibitoren ist insbesondere der im Vergleich zu anderen Lipidsenkern hohe Preis (Stand Februar 2023: Jahrestherapiekosten ca. 6000 €).

#### Gallensäurebinder

Mithilfe von Gallensäurebindern kann der LDL-Cholesterin-Spiegel um bis zu 30 % reduziert werden. Bei Patienten mit vorbestehender Hypertriglyzeridämie können die Triglyzeridwerte ansteigen. Systemische Nebenwirkungen werden nicht beobachtet, da Gallensäurebinder nur im Darm wirken. Sie lösen allerdings gastrointestinale Nebenwirkungen (Stuhlunregelmäßigkeit, Durchfall, Verstopfung, Blähung), die ihren Einsatz beschränken, aus. Daneben können sie mit anderen Medikamente interagieren. Im Rahmen einer älteren Endpunktstudie wurde der klinische Nutzen der Gallensäurebinder bestätigt [25]. Kombinationsstudien mit Statinen liegen nicht vor. Gallensäurebinder sind ein Reservemedikament zur Behandlung der schweren LDL-Hypercholesterinämie.

#### Fibrate

Fibrate führen nur zu einer geringen Reduktion des LDL-Cholesterin-Werts, senken aber die Konzentrationen der Triglyzeride und erhöhen den HDL-Cholesterin-Spiegel [26]. In Monotherapie oder in Kombination mit Nichtstatinlipidsenkern konnte eine kardiovaskuläre Risikoreduktion gezeigt werden [27]. In Kombination mit Statinen fand sich kein positiver Effekt, sodass der Einsatz dieser Medikamentengruppe unter dem Gesichtspunkt der kardiovaskulären Risikoreduktion sehr zurückhaltend gesehen werden sollte [28]. Fibrate können ebenfalls Myopathien auslösen und sind bei eingeschränkter Nierenfunktion (Kreatinin > 3,5 mg/dl, 309 µmol/l; Umrechnung: [mg/dl] • 88,40 = [µmol/l]) kontraindiziert. Zum Einsatz kommen Fibrate bei schwerer isolierter Hypertriglyzeridämie und als Reservemedikament bei Statinunverträglichkeit. In Kombination mit Statinen ist das Risiko für eine Myopathie und Rhabdomyolyse erhöht, wobei darauf geachtet werden sollte, dass nicht Gemfibrozil als Fibrat verabreicht wird, da dessen Myopathierate besonders hoch ist.

#### Omega-3-Fettsäuren

Omega-3-Fettsäuren senken Triglyzeride und erhöhten geringfügig den HDL-Cholesterin-Spiegel. Auf den LDL-Cholesterin-Spiegel haben sie keinen wesentlichen Einfluss. In mehreren Studien konnten niedrig-dosierte Omega-3-Fettsäuren (1 g/Tag) die kardiovaskuläre Ereignisrate nicht beeinflussen und boten keinen Zusatznutzen [29–31]. Im Gegensatz dazu ergab eine Endpunktstudie, dass eine wesentlich höhere Dosis (4 g/Tag) einer spezifischen Omega-3-Fettsäure (Ethylester der Eicosapentaensäure [EPA]) zu einer deutlichen Ereignisreduktion führt [32]. Wodurch der positive Effekt zustande kommt, und ob der Unterschied zu den oben genannten anderen Omega-3-Fettsäure-Studien dadurch bedingt ist, dass ein anderes Patientenkollektiv gewählt wurde, dass eine höhere Dosis eingesetzt wurde, dass Mineralöl als möglicherweise schädliche Vergleichssubstanz oder dass eine spezifische Omega-3-Fettsäure eingesetzt wurde, ist unbekannt.

#### Volanesorsen

Es handelt sich um ein Antisense-Oligonukleotid, das an die mRNA von Apolipoprotein C-III bindet und dessen Produktion verhindert. Es kann Patienten mit familiärem Chylomikronämie-Syndrom und nachgewiesener Mutation verabreicht werden [33]. Als Nebenwirkung können Thrombozytopenien auftreten. Derzeit gibt es in Deutschland nur wenige behandelte Patienten. Im Rahmen der Zulassungsstudien konnte die Rate an Pankreatitiden reduziert werden.

#### Evinacumab

Evinacumab ist ein Antikörper gegen „angiopoietin-like protein 3“ (ANGPTL3), der zu einer Absenkung aller Lipoproteinfraktionen im Plasma führt. Evinacumab ist zurzeit nur zur Behandlung der homozygoten familiären Hypercholesterinämie zugelassen. In dieser Situation können LDL-Cholesterin-Wert-Senkungen um mehr als 50 % beobachtet werden [34].

#### Lomitapid

Lomitapid ist ein Inhibitor des mikrosomalen Transferproteins (MTP) und reduziert die Produktion von ApoB-haltigen Lipoproteinen in Leber und Darm. In der Folge wird in der Blutbahn weniger LDL-Cholesterin gebildet. Da dies unabhängig von den LDL-Rezeptoren erfolgt, ist Lomitapid auch bei homozygoter Familiärer Hypercholesterinämie wirksam und senkt den LDL-Cholesterin-Wert signifikant [35]. Lomitapid ist zurzeit nur zur Behandlung der homozygoten familiären Hypercholesterinämie zugelassen.

#### Lipoprotein(a)-Antisense Oligonukleotid

Mit dem Antisense-Oligonukleotid Lipoprotein(a)-Antisense (Lp[a]-Rx) wird die Bildung von Lipoprotein(a) reduziert. Dosisabhängig können Lipoprotein(a)-Spiegel um bis zu 80 % reduziert werden [37]. Die Substanz wird 4‑wöchentlich s.c. appliziert. Derzeit wird eine große Endpunktstudie durchgeführt, in der überprüft werden soll, ob sich die so induzierte Lipoprotein(a)-Wert-Absenkung in eine kardiovaskuläre Risikoreduktion überträgt. Wenn diese Studie erfolgreich verläuft, könnte der bisher lediglich durch eine Apherese adressierbare unabhängige kardiovaskuläre Risikofaktor Lipoprotein(a) angegangen werden.

##### Merke.

Statine, Ezetimib, Bempedoinsäure und PCSK9-Inhibitoren senken v. a. den LDL-Cholesterin-Spiegel.

### Apherese

Es gibt derzeit eine Reihe von Aphereseverfahren, die den LDL-Cholesterin- und den Lipoprotein(a)-Spiegel um bis zu 70 % absenken können [36]. Diese aufwendigen und teuren Verfahren werden wöchentlich oder 2‑wöchentlich durchgeführt. Zur Absenkung des LDL-Cholesterin-Spiegels kann die Apherese angewendet werden, wenn bei Patienten mit nachgewiesener Atheroskleroseerkrankung das LDL-Cholesterin trotz des Einsatzes aller medikamentösen Maßnahmen deutlich über dem Zielbereich bleibt (insbesondere, wenn die Atheroskleroseerkrankung progredient verläuft). Seit der Verfügbarkeit von PCSK9-Inhibitoren ist die Zahl der Patienten, die wegen erhöhter LDL-Cholesterin-Werte mithilfe der Apherese behandelt werden, deutlich gesunken und spielt nur noch bei ausgeprägter Medikamentenintoleranz und homozygoter familiärer Hypercholesterinämie eine gewisse Rolle.

In Betracht kommt die Apherese auch bei Patienten mit stark erhöhten Lipoprotein(a)-Werten, wenn die Atheroskleroseerkrankung gemäß bildgebender Untersuchungen und klinischer Zeichen progredient verläuft, obwohl alle anderen Risikofaktoren (insbesondere LDL-Cholesterin) optimal eingestellt sind und ein Lipoprotein(a)-Spiegel > 60 mg/dl vorliegt.

## Therapiestrategien

**Figure 1:**
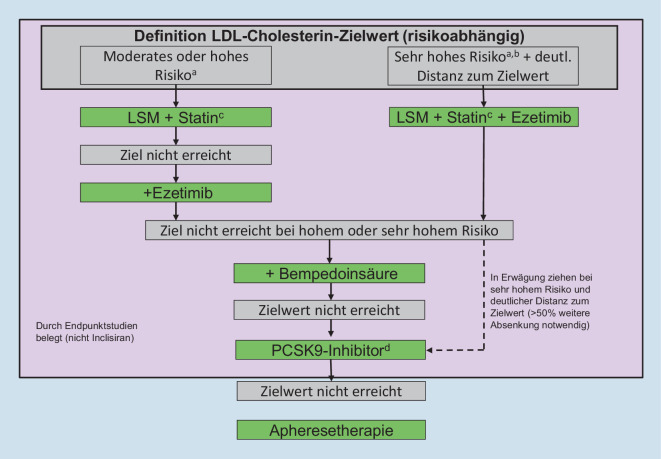

Aufgrund der vorliegenden Endpunktstudien steht die Absenkung des LDL-Cholesterin-Werts in der Prävention kardiovaskulärer Erkrankungen im Vordergrund, unabhängig davon, welche Lipidstoffwechselstörung vorliegt (also LDL-Hypercholesterinämie, kombinierte Hyperlipoproteinämie, Hypertriglyzeridämie oder Lipoprotein[a]-Erhöhung). Welche LDL-Cholesterin-Werte erreicht werden sollen, hängt vom (geschätzten) Absolutrisiko ab (Tab. 2, Abb. 1). Im Folgenden soll dennoch auf die Spezifika der einzelnen Fettstoffwechselstörungen näher eingegangen werden.

### Low-Density-Lipoprotein-Hypercholesterinämie

Lebensstilmaßnahmen spielen zur Absenkung erhöhter LDL-Cholesterin-Werte eine untergeordnete Rolle, da selbst bei konsequenter Umsetzung nur geringe Effekte zu erwarten sind (Absenkung um 5–10 %). In Abhängigkeit vom Absolutrisiko sollte ein LDL-Cholesterin-Zielwert definiert und, wenn nötig, durch den Einsatz von Statinen, Ezetimib, Bempedoinsäure und ggf. PCSK9-Inhibitoren erreicht werden (Abb. 1). In der klassischen Primärprävention (keine Ereignisse, kein Atherosklerosenachweis) ist die Abschätzung über einen Score (z. B. über ESC-Score) hilfreich, um zu evaluieren, ob eine medikamentöse Therapie indiziert ist [3, 38]. Bei einem errechneten Risiko < 1 % für ein tödliches kardiovaskuläres Ereignis in den nächsten 10 Jahren können u. U. auch LDL-Cholesterin-Werte bis 190 mg/dl (4,9 mmol/l) toleriert werden [3].

Der Einsatz von PCSK9-Inhibitoren ist auf Patienten beschränkt, die eine nachgewiesene Atheroskleroseerkrankung haben und den LDL-Cholesterin-Zielwert unter einer Therapie mit Statin plus Ezetimib plus ggf. Bempedoinsäure deutlich verfehlen. Welche Schwelle für den Einsatz eines PCSK9-Inhibitors gewählt wird, ist nicht genau definiert. Je höher das Risiko (z. B. rezidivierende Ereignisse, andere nicht direkt adressierbare Risikofaktoren), desto niedriger die Schwelle (z. B. 90–100 mg/dl), wohingegen bei weniger stark erhöhtem Risiko (z. B. einmaliges Ereignis vor vielen Jahren) auch höhere Schwellenwerte akzeptabel erscheinen. Dies wird unterstützt durch Subgruppenauswertungen der PCSK9-Inhibitorstudien, in denen insbesondere Patienten mit sehr hohem Risiko (z. B. multiple Ereignisse, periphere arterielle Verschlusskrankheit [pAVK], hohe Ausgangswerte etc.) von dieser Therapie profitierten [23]. In Deutschland werden PCSK9-Inhibitoren v. a. bei Patienten mit einer Statinintoleranz eingesetzt [39].

Eine Reihe von Registerstudien der letzten Jahre hat gezeigt, dass selbst Hochrisikopatienten der Zielwert oft nicht erreichen, weil die Therapie nicht entsprechend eskaliert wird [40]. Es stellt sich deshalb die Frage, ob in bestimmten Konstellationen nicht vom sequenziellen Vorgehen abgewichen und eine initiale Kombinationstherapie durchgeführt werden sollte. Ein entsprechender Algorithmus ist in Abb. 1 gezeigt.

Für Patienten mit einer Statinintoleranz stellt die Therapie mit Bempedoinsäure in Kombination mit Ezetimib eine wichtige Option dar. Die Statinintoleranz wird auch in einem separaten Beitrag von Frau Dr. Vogt im vorliegenden Schwerpunktheft abgehandelt.

### Kombinierte Hyperlipoproteinämie

**Figure 2:**
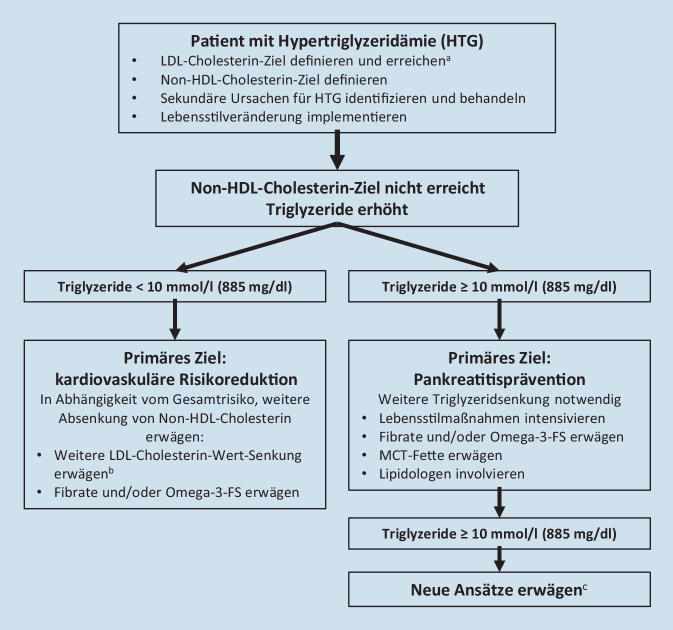

Die kombinierte Hyperlipoproteinämie ist durch eine Konzentrationserhöhung des LDL-Cholesterin-Werts und der Triglyzeride gekennzeichnet. Typischerweise ist der HDL-Cholesterin-Spiegel erniedrigt. Die Konzentrationen des Gesamtcholesterins und das Non-HDL-Cholesterins sind erhöht. Primäres Behandlungsziel stellt wiederum das LDL-Cholesterin dar, wobei sich der Zielwert wieder am Absolutrisiko orientiert (Abb. 2). Wenn nach Erreichen des LDL-Cholesterin-Zielwerts weiter eine Hypertriglyzeridämie besteht (und der sekundäre Zielwert des Non-HDL-Cholesterins nicht erreicht wird), müssen unter Berücksichtigung des Gesamtrisikos ggf. weitere Therapiemaßnahmen erwogen werden. Hierbei sollte die Absenkung des Non-HDL-Cholesterin-Werts im Vordergrund stehen. Dies kann durch eine weitere Absenkung des LDL-Cholesterin-Werts oder durch eine Triglyzerid-Wert-Senkung erfolgen [41]. Hinsichtlich der Triglyzerid-Wert-Senkung stehen Lebensstilmaßnahmen im Vordergrund (Gewichtsabnahme, weitgehender Verzicht auf Alkohol, Reduktion der Zufuhr schnell verstoffwechselbarer Kohlenhydrate).

### Hypertriglyzeridämie

Bei der isolierten Hypertriglyzeridämie ist neben den Triglyzeridkonzentrationen oft auch der Non-HDL-Cholesterin-Spiegel erhöht (Abb. 2). Unter dem Gesichtspunkt der Atheroskleroseprävention sollte auch hier primär der LDL-Cholesterin-Spiegel adressiert werden, wobei oft weniger das Erreichen des Absolutziels als die Absenkung um mindestens 50 % im Vordergrund steht. Auch hier kommen primär Statine und Ezetimib zum Einsatz. Eine Absenkung erhöhter Triglyzeridspiegel sollte ggf. unter den bei der „kombinierten Hyperlipoproteinämie“ genannten Gesichtspunkten erfolgen.

Bei hohen Triglyzeridwerten (meist > 1000 mg/dl; ca. 10 mmol/l) kann es zum Auftreten einer akuten Pankreatitis (Chylomikronämiesyndrom) kommen. Interessanterweise zeigen epidemiologische Daten, dass das Pankreatitisrisiko bereits bei gering erhöhten Triglyzeridwerten ansteigt (in absoluten Zahlen aber sehr gering ist: 2,7 Ereignisse/10.000 Personenjahren bei Triglyzeridwerten < 1 mmol/l; 5,5 Ereignisse/10.000 Personenjahren bei Triglyzeriden 2,0–3,0 mmol/l; „hazard ratio“ [HR] 1,8); [11]. Besonders hoch ist das Pankreatitisrisiko beim familiären Chylomikronämiesyndrom [42].

Bei dauerhaft erhöhten Triglyzeridwerten über 400–600 mg/dl (4,5–6,8 mmol/l) sollte unter dem Einsatz von Lebensstilmaßnahmen, Fibraten und/oder Omega‑3 Fettsäuren versucht werden, die Triglyzeridwerte unter 400 mg/dl einzustellen, um das Risiko für eine akute Pankreatitis zu reduzieren. Tritt eine hypertriglyzeridämieinduzierte Pankreatitis auf, werden als Basismaßnahmen eine strikte Nahrungskarenz und eine i.v.-Flüssigkeitssubstitution durchgeführt. Es kann individuell entschieden werden, ob eine Plasmapherese erfolgen werden soll, da es hierzu keine randomisierten Studien gibt.

Für Patienten mit sehr seltenen angeborenen schweren Formen der Hypertriglyzeridämie (familiäres Chylomikronämiesyndrom z. B. bei Lipoprotein-Lipase-Defekt) steht als neuer Therapieansatz das ApoC3-Antisense-Oligonukleotid Volanesorsen zur Verfügung. Volanesorsen kann die Triglyzeridspiegel deutlich reduzieren und das Pankreatitisrisiko vermindern [33].

### Lipoprotein(a)-Hyperlipoproteinämie

Derzeit gibt es keine medikamentösen Möglichkeiten oder Lebensstilmaßnahmen, um erhöhte Lipoprotein(a)-Spiegel abzusenken. Die Optimierung des Gesamtrisikoprofils steht deshalb im Vordergrund. Dies beinhaltet auch die optimale Einstellung des LDL-Cholesterin-Spiegels, ggf. unter Verwendung von Statinen, Ezetimib, Bempedoinsäure und PCSK9-Inhibitoren. Letztere können auch den Lipoprotein(a)-Spiegel etwas absenken, sind aber nicht zur Behandlung der Lipoprotein(a)-Konzentrationserhöhung zugelassen. Bei Patienten, bei denen trotz einer über länger als ein Jahr guten Einstellung aller anderen Risikofaktoren und eines Lipoprotein(a)-Spiegels über 60 mg/dl klinische Zeichen und bildgebende Untersuchungen auf eine progrediente Atheroskleroseerkrankung hinweisen, kann eine regelmäßige Apheresetherapie in Erwägung gezogen werden [36].

Da Patienten mit erhöhten Lipoprotein(a)-Werten häufig eine positive Familienanamnese aufweisen, sollten ähnlich wie bei der familiären Hypercholesterinämie erstgradig Verwandte dahingehend untersucht werden.

## Fazit für die Praxis

- Die Behandlung von erhöhten Lipidwerten spielt in der Atheroskleroseprävention eine zentrale Rolle; hierbei kommt der LDL-Cholesterin-Wert-Senkung mit Statinen und, wenn nicht ausreichend, mit Ezetimib, Bempedoinsäure und Inhibitoren der Proproteinkonvertase Subtilisin/Kexin Typ 9 (PCSK9) die größte Bedeutung zu.
- Lebensstilmaßnahmen können das kardiovaskuläre Risiko stark beeinflussen, spielen aber für die Absenkung des LDL-Cholesterin-Werts nur eine untergeordnete Rolle.
- Für die Entscheidung, ob, und ggf. wie intensiv, eine Lipidsenkung durchgeführt werden soll, ist das kardiovaskuläre Absolutrisiko entscheidend. Die Zielwerte sind in den letzten Jahren aufgrund der Ergebnisse von Interventionsstudien weiter abgesenkt worden.
- Lebensstilmaßnahmen können Triglyzeride deutlich absenken und sind oft effektiver als spezifische Triglyzeridsenker wie Fibrate und Omega-3-Fettsäuren.
- Neue Lipidsenker zur Behandlung bei stark erhöhten Triglyzerid- und erhöhten Lipoprotein(a)-Werten sind in der Entwicklung, müssen ihren Nutzen aber erst in Endpunktstudien belegen.
