# Erratum zu: Update Lipidologie

**DOI:** 10.1007/s00108-023-01641-8

**Published:** 2024-01-03

**Authors:** Klaus G. Parhofer

**Affiliations:** Medizinische Klinik IV – Großhadern, Klinikum der Universität München, Marchioninistr. 15, 81377 München, Deutschland

**Erratum zu:**

**Innere Medizin 2023**

10.1007/s00108-023-01536-8

Der Artikel *Update Lipidologie* von Klaus G. Parhofer wurde ursprünglich Online First ohne Open Access auf der Internetplattform des Verlags publiziert. Nach der Veröffentlichung in Bd. 64, Heft 7, pp. 611–621 hatten sich die Autoren für eine Open-Access-Veröffentlichung entschieden. Das Urheberrecht des Artikels wurde deshalb in © Der/die Autor(en) 2023 geändert. Dieser Artikel ist jetzt unter der Creative-Commons-Namensnennung 4.0 International-Lizenz veröffentlicht, welche die Nutzung, Vervielfältigung, Bearbeitung, Verbreitung und Wiedergabe in jeglichem Medium und Format erlaubt, sofern Sie den/die ursprünglichen Autor(en) und die Quelle ordnungsgemäß nennen, einen Link zur Creative-Commons-Lizenz beifügen und angeben, ob Änderungen vorgenommen wurden.

Die in diesem Artikel enthaltenen Bilder und sonstiges Drittmaterial unterliegen ebenfalls der genannten Creative-Commons-Lizenz, sofern sich aus der Abbildungslegende nichts anderes ergibt. Sofern das betreffende Material nicht unter der genannten Creative-Commons-Lizenz steht und die betreffende Handlung nicht nach gesetzlichen Vorschriften erlaubt ist, ist für die oben aufgeführten Weiterverwendungen des Materials die Einwilligung des jeweiligen Rechteinhabers einzuholen.

Weitere Details zur Lizenz entnehmen Sie bitte der Lizenzinformation auf http://creativecommons.org/licenses/by/4.0/deed.de.

